# Genetic Predisposition to Hippocampal Atrophy and Risk of Amnestic Mild Cognitive Impairment and Alzheimer’s Dementia

**DOI:** 10.3390/geriatrics10010014

**Published:** 2025-01-16

**Authors:** Ioannis Liampas, Vasileios Siokas, Niki Mourtzi, Sokratis Charisis, Stefanos N. Sampatakakis, Ioannis Foukarakis, Alex Hatzimanolis, Alfredo Ramirez, Jean-Charles Lambert, Mary Yannakoulia, Mary H. Kosmidis, Efthimios Dardiotis, Georgios M. Hadjigeorgiou, Paraskevi Sakka, Konstantinos Rouskas, Nikolaos Scarmeas

**Affiliations:** 1 Department of Neurology, University Hospital of Larissa, School of Medicine, University of Thessaly, 41100 Larissa, Greece; iliampas@uth.gr (I.L.); vsiokas@med.uth.gr (V.S.); edar@med.uth.gr (E.D.); 2 1st Department of Neurology, Aiginition Hospital, National and Kapodistrian University of Athens Medical School, 11528 Athens, Greece; nikimourtzi23@gmail.com (N.M.); scharissis@gmail.com (S.C.); stefanos.sab@gmail.com (S.N.S.); jfoukarakis@gmail.com (I.F.); 3 Department of Neurology, UT Health San Antonio, San Antonio, TX 78229, USA; 4 Department of Psychiatry, Aiginition Hospital, National and Kapodistrian University of Athens Medical School, 11528 Athens, Greece; alhatzi@gmail.com; 5 Division of Neurogenetics and Molecular Psychiatry, Department of Psychiatry and Psychotherapy, Medical Faculty, University of Cologne, 50923 Cologne, Germany; alfredo.ramirez@uk-koeln.de; 6 Department of Neurodegenerative Diseases and Geriatric Psychiatry, University Hospital Bonn, 53127 Bonn, Germany; 7 German Center for Neurodegenerative Diseases (DZNE Bonn), 53175 Bonn, Germany; 8 Department of Psychiatry, Glenn Biggs Institute for Alzheimer’s and Neurodegenerative Diseases, San Antonio, TX 78229, USA; 9 Excellence Cluster on Cellular Stress Responses in Aging-Associated Diseases (CECAD), University of Cologne, 50931 Cologne, Germany; 10 U1167-RID-AGE Facteurs de Risque et Déterminants Moléculaires des Maladies Liés au Vieillissement, CHU Lille, Inserm, Institut Pasteur de Lille, Université de Lille, 59000 Lille, France; jean-charles.lambert@pasteur-lille.fr; 11 Department of Nutrition and Dietetics, Harokopio University, 17671 Athens, Greece; myianna@hua.gr; 12 Lab of Cognitive Neuroscience, School of Psychology, Aristotle University of Thessaloniki, 54124 Thessaloniki, Greece; kosmidis@psy.auth.gr; 13 Department of Neurology, Medical School, University of Cyprus, Nicosia 2414, Cyprus; hadjigeorgiou.georgios@ucy.ac.cy; 14 Athens Association of Alzheimer’s Disease and Related Disorders, 11636 Maroussi, Greece; info@psakka.gr; 15 Institute of Applied Biosciences, Centre for Research & Technology Hellas, 54124 Thessaloniki, Greece; rouskas@certh.gr; 16 Department of Neurology, The Gertrude H. Sergievsky Center, Taub Institute for Research in Alzheimer’s Disease and the Aging Brain, Columbia University, New York, NY 10032, USA

**Keywords:** hippocampal atrophy, Alzheimer’s disease, cognitive decline, polygenic risk score

## Abstract

Background: There is a paucity of evidence on the association between genetic propensity for hippocampal atrophy with cognitive outcomes. Therefore, we examined the relationship of the polygenic risk score for hippocampal atrophy (PRShp) with the incidence of amnestic mild cognitive impairment (aMCI) and Alzheimer’s disease (AD) as well as the rates of cognitive decline. Methods: Participants were drawn from the population-based HELIAD cohort. Comprehensive neuropsychological assessments were performed at baseline and at follow-up. PRShp was derived from the summary statistics of a large genome-wide association study for hippocampal volume. Cox proportional hazards models as well as generalized estimating equations (GEEs) were used to evaluate the association of PRShp with the combined incidence of aMCI/AD and cognitive changes over time, respectively. All models were adjusted for age, sex, education, and apolipoprotein E (APOE) genotype. Results: Our analysis included 618 older adults, among whom 73 developed aMCI/AD after an average follow-up of 2.96 ± 0.8 years. Each additional SD of PRShp elevated the relative hazard for incident aMCI/AD by 46%. Participants at the top quartile of PRShp had an almost three times higher risk of converting to aMCI/AD compared to the lowest quartile group. Higher PRShp scores were also linked to steeper global cognitive and memory decline. The impact of PRShp was greater among women and younger adults. Conclusions: Our findings support the association of PRShp with aMCI/AD incidence and with global cognitive and memory decline over time. The PRS association was sex- and age-dependent, suggesting that these factors should be considered in genetic modelling for AD.

## 1. Introduction

The amyloid–tau–neurodegeneration (ATN) framework reflects the pathological hallmarks of Alzheimer’s disease (AD): Aβ amyloid deposition (A), tau aggregation (T), and neurodegeneration (N). These markers differentiate AD pathology from other neurocognitive disorders [1]. Unfortunately, the evaluation of ATN markers is limited by the cost or due to the invasive nature of the associated procedures.

Of note, novel findings from genetic association studies suggest that AD-related pathology is strongly linked to genetics, leading to a deeper understanding of how genetics contribute to AD-related neurodegeneration [2,3]. Although genetic measures (such as polygenic risk score (PRS)) fail to encapsulate the full and changing landscape of neurodegeneration, they present important advantages in that they capture the time-invariant risk-conferring constituents of AD-related pathology (regardless of the age and time of assessment) and provide better large-scale applicability. There is, however, a paucity of published evidence on the direct association between genetics and clinical outcomes, as the majority of studies have focused on endophenotypes rather than on clinical manifestations [4,5].

Hippocampal volume is a well-established structural marker of AD-related neurodegeneration [6]. Numerous studies have already related hippocampal atrophy to more precipitous trajectories of cognitive decline and greater risk of incident AD [7,8]. Of note, hippocampal volumes are highly heritable, with genetic factors accounting for 50–70% of the variability in hippocampal size [4,9]. In a genome-wide association study (GWAS), genes linked to neuronal differentiation, locomotive and exploratory behavior, AD, and schizophrenia were associated with hippocampal subfield volumes; the reported SNP-based heritability estimates ranged between 0.1 to 0.3 [10]. However, to our knowledge, only one study has found an association between genetic propensity for higher hippocampal volumes with better executive function and slower verbal fluency decline [4].

Therefore, the aim of the current study was to explore the longitudinal association of genetic predisposition towards smaller hippocampal volumes (hippocampal atrophy) with (a) the incidence of Alzheimer’s disease dementia (AD) or amnestic mild cognitive impairment (aMCI); and (b) rates of cognitive decline. For this purpose, we capitalized on data from the population-based HELIAD (Hellenic Longitudinal Investigation of Aging and Diet) cohort, a randomly selected sample of community-dwelling older adults. The PRS for hippocampal volume (PRShp) was derived from the summary statistics of a large GWAS [10]. The potential dependence of genetic risk for hippocampal atrophy on age and sex was also examined.

## 2. Materials and Methods

### 2.1. Settings and Participants

Our sample was drawn from the prospective HELIAD study, a multidisciplinary population-based cohort that primarily explores the epidemiology of dementia and other neuropsychiatric disorders in an older Greek population [11]. Study procedures were approved by the Institutional Ethics Review Boards of the National and Kapodistrian University of Athens and the University of Thessaly and were conducted in accordance with principles of good clinical practice and the Helsinki Declaration or later amendments. All participants or surrogates provided informed consent prior to participation.

Older participants (>64 years) were randomly selected from the rosters of two Greek municipalities, one in the rural area of Larissa (in the province of Thessaly) and one in the urban area of Marousi (a suburb of Athens). Collaborative assessments were conducted by certified neurologists, trained neuropsychologists, and dieticians. Relevant information was collected either from participants themselves or from participants’ carers (first-degree relatives, spouses, etc.), whenever it was deemed necessary. Participants underwent baseline and follow-up evaluations at approximately 3-year intervals. Extensive details about the design and key features of the HELIAD study have been described previously [12].

### 2.2. Neuropsychological Evaluation

Cognition was evaluated by trained neuropsychologists according to a designated approach involving the comprehensive assessment of all major cognitive domains [13]. Episodic memory was assessed through the Greek verbal learning test (GVLT; verbal memory) and the Medical College of Georgia Complex Figure Test (MCG; non-verbal memory). Language was appraised on the semantic and phonological fluency tasks, subtests of the Greek version of the Boston Diagnostic Aphasia Examination short form (BDAE; the Boston Naming Test—short form), and selected items from the Complex Ideational Material Subtest to assess verbal comprehension and repetition of words and phrases. Visuospatial ability was assessed using the Judgment of Line Orientation abbreviated form, the MCG copy condition, and the clock drawing test. Attention and processing speed were evaluated using the Trail Making Test—Part A (TMT-A). Executive functioning was assessed using the Trail Making Test—Part B (TMT-B), anomalous sentence repetition (created for the present investigation), a graphical sequence test, and a motor programming test. Individual test raw scores were converted into z-scores using mean and standard deviation (SD) values of the participants who were cognitively normal (without MCI or dementia) at baseline [14,15,16]. In turn, z-scores from individual tests were averaged to produce domain z-scores for episodic memory, language, attention, executive function, and visuo-perceptual ability. Domain z-scores were averaged to produce a z-score for global cognition. Higher scores were consistent with better cognitive performance.

### 2.3. Diagnostic Approach

All participants underwent a standard physical and neurological examination. A particular focus was placed on identifying potential comorbidities that could interfere with cognition through screening the participants for depression, anxiety, essential tremor, behavioral symptoms, Parkinson’s disease, dementia with Lewy bodies (DLB), and a personal history of cerebrovascular disease accounting for the onset or deterioration of cognitive decline. Information was also gathered on comorbidities, regular medication intake, sleep and dietary habits, mental and physical activity, as well as laboratory tests (imaging studies and blood examinations), when available.

The diagnostic classification of the participants according to their cognitive status was established during expert consensus meetings involving senior neurologists and neuropsychologists. Dementia and AD were diagnosed according to the Diagnostic and Statistical Manual of Mental Disorders -IV-text revision criteria [17] and the National Institute of Neurological and Communicative Disorders and Stroke/Alzheimer Disease and Related Disorders Association criteria [18], respectively. The diagnosis of vascular dementia was based on a history or clinical evidence of stroke, the presence of a clear temporal relation between stroke and the onset of dementia, and the Hachinski Ischemia Scale score [19]. Lewy body and frontotemporal dementias were diagnosed based on the respective criteria [20,21]. MCI and its subtypes were diagnosed according to the Petersen criteria [22]. MCI was categorized as amnestic in cases of isolated memory impairment or multi-domain impairment involving memory, and as non-amnestic in cases of isolated or combined language, attention, executive, or visuo-perceptual impairment (not involving memory).

### 2.4. Genotyping and Imputation

Genome-wide genotyping was performed at Life & Brain GmbH facilities (Bonn 53127, Germany) using the Illumina Infinium Global Screening Array (GSA, GSAsharedCUSTOM_24 + v1.0) and calling was generated by the “centre national de recherche en génétique humaine” (Evry, Essone, France) using the data generated by the centers involved in genotyping (Life & Brain GmbH, CNRGH, and Amsterdam). Details have been described elsewhere (Supplementary Materials S1.1) [23].

### 2.5. Polygenic Risk Score Calculation

The PRS for hippocampal volume was derived from the summary statistics of a large GWAS on the genetic architecture of hippocampal volumes. The data for this study were derived from 21,297 participants across 16 different cohorts who had T1-weighted brain scans available [10]. In the GWAS summary statistics, each SNP is associated with hippocampal volume levels at a certain *p*-value threshold.

Details on the polygenic risk calculation are included in Supplementary Materials S1.2. For each subject, we computed 10 different genome-wide PRSs for hippocampal volumes based on an a priori set of 10 *p*-value thresholds (P_T_). To calculate each PRS, we consecutively used reduced sets of genetic variants, summing over all SNPs meeting each of the following significance thresholds: *p* < 0.0001; *p* < 0.001; *p* < 0.01; *p* < 0.05; *p* < 0.1; *p* < 0.2; *p* < 0.3; *p* < 0.4; *p* < 0.5; and *p* < 1.0. Given that hippocampal volumes are inversely related to the risk of incident AD, PRSs for hippocampal volumes were multiplied by −1 to align a higher risk score with smaller hippocampal volumes. To facilitate interpretation, PRSs were standardized to have a mean of zero and an SD of 1.

Since each PRS threshold consists of a different set of SNPs, we constructed a set of logistic regression models with the presence of AD–aMCI as the outcome and the different PRS thresholds as the main predictors. Then, receiver operator characteristic (ROC) curves were constructed for each model. The area under the curve (AUC) and *p*-values were estimated. The PRS threshold of the model with the best classification accuracy, estimated by calculating the area under the ROC curve, was considered as the one that more accurately predicted hippocampal volumes and was thus used as the measure of genetic predisposition for smaller hippocampal volumes (Table S1).

### 2.6. Statistical Analyses

Analyses were performed using SPSS 26 (SPSS, Chicago, IL, USA). Baseline characteristics between groups were compared via independent sample t-test for continuous variables and Pearson’s chi-squared test for categorical variables. The significance level was set at α ≤ 0.05.

PRSs were normally distributed according to the Kolmogorov–Smirnoff and Shapiro–Wilk tests for normality; therefore, we initially treated the standardized PRS as a scale variable. In turn, to evaluate potential threshold effects we trichotomized our cohort according to PRS quartiles into low (1st quartile) vs. intermediate (2nd and 3rd quartiles) vs. high (4th quartile) PRSs. Adjusted models included age and education (years of formal schooling) as covariates in a continuous scale, together with sex (female vs. male as reference) and *ApoE* genotype (*ApoE4* carriers vs. *ApoE4* non carriers as reference), which were treated as categorical variables. To correct for cryptic relatedness between subjects or unexpected genotyping batch-related errors [24], we included the first two principal components (PCs) of the whole sample as covariates in the models, similar to methods used in previous studies [5].

#### 2.6.1. Cox Proportional Hazard Models

In Cox models, the combined incidence of clinical aMCI (among all MCI types) and AD (among all dementia types) was used as a dichotomous outcome. We used this composite event due to the substantially small number of incident AD cases that would considerably undermine the power our analyses. We selected aMCI and no other MCI types because aMCI has been defined as a prodromal stage of AD, since a considerably higher rate of aMCI cases will be converted to AD compared to other types of MCI. Participants with a baseline diagnosis of dementia or aMCI were removed from the analysis. Participants that converted to other dementia entities at follow-up were excluded as well, due to the competing nature of different dementia diagnoses. Those who did not develop aMCI or dementia were censored at follow-up. The interval between the baseline evaluation and follow-up was inserted as the time-to-event variable.

First, we examined the association of PRS for hippocampal atrophy with risk of aMCI or AD over time through Cox models adjusted for age, sex, educational attainment, *ApoE* genotype, PC1 and PC2. The proportionality of hazards assumption was confirmed using Cox regression analyses with time-dependent covariates. In particular, for each PRS, an extended Cox model including the term PRS*time along with the PRS was analyzed. To verify that the proportionality of hazards assumption was not violated, the coefficient of the time interaction product was required to be statistically insignificant. Afterwards, the proportionality of hazards for different PRS strata (quartile approach) over time was confirmed via the same approach.

Then, we performed subgroup analyses to examine for potential sex and age cohort effects [5], as follows: (1) men vs. women and (2) according to age. Participants were divided into older vs. younger participants using the median of our sample as a cut-off (72.67 years).

#### 2.6.2. Generalized Estimating Equations

The effect of the PRS for hippocampal atrophy on rates of cognitive decline was explored using generalized estimating equations (GEE) analyses. GEE accounts for the potential correlation of repeated measurements in the same individual. We treated each participant’s baseline and follow-up evaluations as a cluster. Both autoregressive and exchangeable covariance matrices were used as working correlation structures with comparable results. Six consecutive GEE models were explored using the composite and individual domain cognitive measurements (memory, language, executive function, visuospatial perception, and attention) as the dependent scale variables. GEE analyses featured the main effects of the PRS (first as a scale variable and then as a trichotomous variable) and time from baseline as well as the PRS by time interaction terms. Models were again controlled for age, educational attainment, sex, *ApoE* genotype, PC1, and PC2.

Finally, to explore for potential disparities regarding the impact of the PRS on differences in sex and age, we performed subgroup analyses based on the same approach described above.

## 3. Results

### 3.1. Participant Characteristics and Missing Data Analysis

There were 1017 unrelated, older adults with available genetic data and without aMCI or dementia at baseline. Follow-up information was not available for 378 participants; 52 converted to aMCI; 21 progressed to AD; 4 developed other dementias; and 16 had missing follow-up values on MCI–dementia. Thus, the present analysis involved a total of 619 participants at baseline, 73 of whom developed aMCI or AD at follow-up. Compared to those included (*n* = 619), those without available follow-up information (*n* = 378) were older (74.7 ± 5.3 vs. 73.4 ± 5.0 years, *p* < 0.001) and less educated (6.5 ± 4.1 vs. 7.3 ± 4.7 years, *p* = 0.002). Sex (*p* = 0.612) and ApoE_4_ (*p* = 0.659) distributions were similar between the two groups. No differences were found with respect to PRS.

The average follow-up of included participants was 2.96 ±0.80 years (between 1.28 and 6.93 years). The demographic and genetic characteristics of the total sample, based on the incidences of aMCI or AD, are presented in Table 1. Those who developed aMCI or AD at follow-up were older, less educated, and had a higher PRS for hippocampal atrophy.

### 3.2. Polygenic Risk Score for Hippocampal Atrophy and Incident Amnestic MCI or AD

The proportionality of hazards assumption was confirmed for both scale and trichotomous PRSs. First, we tested the unadjusted relationship between the ten PRSs constructed at different PTs and the risk of aMCI or AD (Supplementary Table S1). Results showed a significant unadjusted association between the PRS calculated at PT = 0.01 (8475 SNPs included) and aMCI–AD status at follow-up. Higher PRS values were associated with a significantly higher hazard for progressing to aMCI or AD; one SD increased the relative risk by 39% (*p* = 0.005). When participants were trichotomized according to PRS quartiles, higher PRS strata were again related to significantly higher aMCI–AD risk at the 0.01 PT (*p* for trend = 0.036). Results showed that those who belonged to the highest quartile of the PRS for hippocampal atrophy had ~2.57 higher risk of developing aMCI or AD (*p* = 0.011) compared to individuals belonging to the bottom quartile of the PRS.

**Figure 1:**
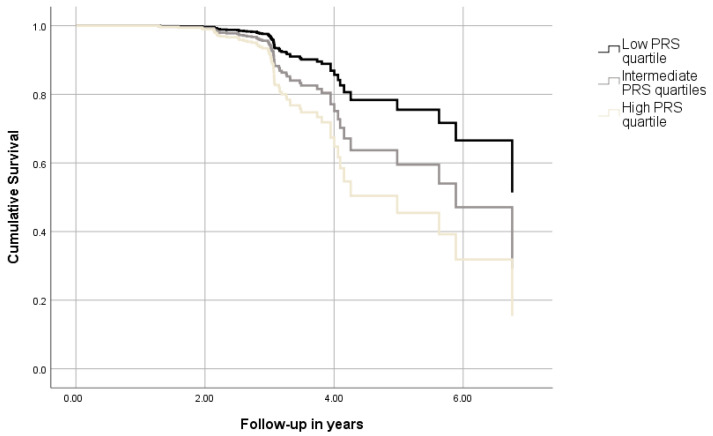
Cumulative survival curves depicting the combined aMCI–AD risk of participants with different levels of PRShp (*p* for trend = 0.021). The figure was derived from a model adjusted for age, sex, education, PC1, PC2, and APOE ε4 genotype.

Subsequently, adjusted Cox models were tested using the PRS calculated at PT = 0.01 (Table 2). Adjusted associations were even more prominent, with one SD elevating the relative hazard for incident aMCI or AD by 46% (*p* = 0.002). At the same time, the top quartile of the PRS exhibited almost three (~2.81, *p* = 0.006) times higher risk of progressing to aMCI or AD compared to the lowest quartile (Figure 1).

In subgroup analyses (Table 2), it was revealed that the association of the PRS for hippocampal atrophy with incident aMCI–AD was mostly driven by women, with one SD of the PRS enhancing the relative hazard by 60% in women. In parallel, the upper PRS quartile had almost three times (~2.89, *p* = 0.033) the risk of developing aMCI or AD at follow-up, compared to the lowest quartile. By contrast, the results were not significant in men. Regarding age, genetic propensity for hippocampal atrophy was found to affect younger individuals more than older ones. Specifically, one additional SD of the PRS increased the risk of future aMCI–AD by almost two (~1.87) times in those younger than 72.67 years, while associations did not achieve statistical significance in the older age group.

### 3.3. Polygenic Risk Score for Hippocampal Atrophy and Rates of Cognitive Decline

First, we tested adjusted GEE models using the PRS as a scale variable (Table 3). The results suggested that for each extra SD of the PRS, the rates of yearly global cognitive decline increased by 1.3% for each SD. Although comparable associations were captured for memory (1.5%), executive function (1.2%), and language (1.2%), these estimates were less precise and did not reach statistical significance. Subsequently, we tested the adjusted GEE models using the PRS as a trichotomous variable (Table 3). It was found that the top PRS quartile experienced more precipitous memory (by 5.1% per year) and global cognitive decline (by 3.8% annually) compared to the low quartile.

In subgroup analyses, it was revealed once again that the relationship between genetic predisposition to hippocampal atrophy and cognitive decline was mostly driven by women (Supplementary Table S1). In specific, a higher PRS was related to steeper global cognitive, executive, memory, language, as well as visuo-perceptual decline in older female, but not male, adults. The association between PRS and cognitive decline was differentiated only with respect to executive function between younger and older adults; executive function diminished in a steeper fashion among those younger than 72.67 years (Supplementary Table S2).

## 4. Discussion

In the present study, we found that the PRS for hippocampal atrophy was related to a greater risk of aMCI or AD, as well as to steeper global cognitive and memory declines. It was revealed that these associations were more prominent in women compared to men and in younger versus older adults. Of note, in the female subgroup, apart from memory, a higher PRS was associated with steeper global cognitive, executive function, language, and visuo-perceptual skills declines.

The hippocampus is the central hub of a complex brain network supporting episodic memory [25]. Episodic memory impairment is the neuropsychological correlate of typical AD, while aMCI (involving episodic memory impairment) is the MCI precursor of AD [26,27]. Initial pathological alterations in AD take place in the medial temporal lobes, including the hippocampal formations [28]. Subsequently, hippocampal volume loss is an early structural change in cognitively unimpaired (CU) individuals at high risk of developing AD and is considered a specific marker of AD-related neurodegeneration [29]. As AD-related pathology accumulates, the brain reserve—a term that encompasses the structural characteristics of the brain that enable individuals to better cope with neuropathological alterations—assumes a crucial role in an individual’s susceptibility to disease [30]. In this general framework, the genetic predisposition towards hippocampal atrophy could not only reflect an increased propensity for AD-related neurodegeneration but also to decreased resilience, leading to an unsuccessful adaptation to these changes.

Amyloid-β deposition has been longitudinally related to hippocampal volume loss spreading from selective subfields across the hippocampus and concurring memory decline [31]. Recent evidence suggests that large hippocampal volumes may represent a measure of brain reserve that allows older adults to maintain normal cognition in spite of amyloid accumulation [32]. According to this scenario, in the presence of comparable pathological changes, individuals with larger hippocampal volumes have greater brain resilience, will remain asymptomatic for longer periods of time, and progress towards aMCI–AD in a slower manner. However, until additional evidence is acquired, the concept that hippocampal volumes constitute a measure of brain reserve will remain purely theoretical.

Irrespective of the neurobiological underpinnings, hippocampal atrophy is an early structural change in CU individuals at high risk of developing AD, and is considered a specific marker of AD-related neurodegeneration [29]. Structural magnetic resonance imaging (MRI) is the standard modality used to quantify hippocampal volumes. The routine utilization of structural MRIs, especially in cases of CU older adults (for research purposes) is hindered by a number of limitations. The PRS for hippocampal atrophy admittedly outweighs the standard modality in terms of procedure-related limitations (e.g., tolerability or applicability in cases of metal implants), re-evaluation requirements (PRS values are interchangeable regardless of the age and timing of assessment), and large-scale applicability. Hippocampal volumes constitute a highly heritable trait [4,9] and our findings suggest that a PRS for hippocampal atrophy could be useful in a research setting as a potential alternative to the structural MRI in studies conducted on large cohorts with available genetic data such as the UK Biobank [33].

Of note, the impact of the PRS was found to be sex-dependent and more pertinent to women. The neurobiology of cognition differs between men and women, with sex differences likely being influenced by a great number of factors, including psychosocial, cultural, behavioral, and, most notably, biological parameters (e.g., sex hormones). Previous research examining sex interactions with AD-related pathology has demonstrated that the long-standing female advantage in episodic memory and verbal fluency (which is heavily based on executive skills) is moderated by, or may even vanish in, the presence of biomarkers of AD-related pathology in non-demented adults (e.g., positive amyloid positron emission tomography, high amyloid level in the cerebrospinal fluid, lower temporal lobe glucose metabolic rates or smaller hippocampal volumes) [34,35,36]. Notably, a published survey has specifically found that the female supremacy in episodic memory is diminished with smaller hippocampal volumes among individuals with aMCI [37]. In line with these findings, and using the PRS for hippocampal atrophy as a proxy for smaller hippocampal volumes, we found that greater hippocampal atrophy is related to steeper cognitive changes in female, compared to male, older adults. Further studies should examine if this association could in time moderate or eliminate the long-standing female advantage in cognition.

As for age, although previous research has shown that hippocampal volumes are longitudinally related to cognitive—especially episodic memory—changes over time in older adults above 65 years of age, potential differences between younger versus older individuals in this age group have not been explored [38]. Therefore, the reproducibility of our findings cannot be tested.

### Strengths and Limitations

The present article has several strengths. To our knowledge this is the first study examining the association between the PRS for hippocampal atrophy and incidences of aMCI and AD. Subgroup analyses were performed to identify the potential exaggerated (or deflated) influence of genetic predisposition to hippocampal atrophy in different demographic subgroups. Another strength is the longitudinal design of our study, which enabled us to eliminate any life-long genetic effects on our subgroup of older individuals and focus on the contemporary impact of genetics during the follow-up period of the study. To address the potential impact of advanced, ongoing neurodegenerative processes on the rates of cognitive decline we analyzed only participants with normal cognition throughout the follow-up. An expert-consensus, clinically established diagnosis of dementia based on standard criteria and supported by a comprehensive neuropsychological evaluation augmented the accuracy of the diagnostic categorization of the participants.

However, the current survey also has several limitations. First, volumetric measurements from MRI scans were not available and thus information on the intermediate phenotype was lacking. Second, we did not have access to ATN biomarkers to corroborate the clinically established neuropsychiatric diagnoses; such biomarkers are considered to increase precision in the identification and differential diagnosis of dementia. Therefore, a misclassification bias may yet be present. Third, non-response and attrition biases may have influenced our findings, as follow-up assessments were not conducted for a non-trivial proportion of the original sample. Additionally, the moderate follow-up period of the present study led to the documentation of a small number of events and may have underpowered our analyses. Moreover, HELIAD participants are of Greek ancestry and the results may not be applicable to other ethnic groups. Further studies should be performed in other ethnic groups to confirm these findings. Finally, we must acknowledge that the original GWAS on the genetic architecture of hippocampal volumes was based on T1-weighted MRI studies [10]. More sophisticated structural neuroimaging or a combination of T1- and T2-weighted images might better capture morphological alterations. Of note, neuropsychological tasks may well be adapted to functional MRI paradigms. Functional rather than structural alterations may constitute earlier and more specific neuroimaging correlates of cognitive performance. Hopefully, the methodological limitations hindering the replicability of functional MRI study findings will be overcome in the near future [39].

## 5. Conclusions

The PRS for hippocampal atrophy is related to greater risk of incident aMCI or AD, as well as to steeper cognitive decline. These associations are more prominent among women and younger adults above the age of 65. Considering the high heritability of hippocampal volumes, future studies ought to confirm that the PRS may serve as a proxy biomarker for hippocampal atrophy.

## Figures and Tables

**Table 1:** Participants’ baseline clinical and genetic characteristics by incidence of aMCI–AD.

Parameters	Total Sample	Non aMCI ^1^/AD ^2^ at Follow-Up	Amci ^1^/AD ^2^ at Follow-Up	
	N = 619	N = 546	N = 73	*p*-Value
Age (years), mean ± SD	73.4 ± 5.0	73.1 ± 4.9	75.1 ± 5.4	**0.001**
Sex, female (%)	361 (58.3%)	321 (58.8%)	40 (54.8%)	0.515
Education (years), mean ± SD	7.3 ± 4.5	7.5 ± 4.5	5.8 ± 4.3	**0.002**
ApoE ε4 carrier, *n* (%)	98 (15.8%)	86 (15.8%)	12 (16.4%)	0.880
PRShp ^3^, mean ± SD	−0.01 ± 0.98	−0.05 ± 0.98	0.33 ± 0.96	**0.002**
PRS strata using quartiles				
High	154 (24.9%)	128 (23.4%)	26 (35.6%)	**0.018**
Intermediate	311 (50.2%)	274 (50.2%)	37 (50.7%)	
Low	154 (24.9%)	144 (26.4%)	10 (13.7%)	

^1^ amnestic mild cognitive impairment, ^2^ Alzheimer’s disease, ^3^ polygenic risk score. Scale variables are presented in mean ± SD; categorical variables are presented as absolute numbers (percentages); *p*-values refer to differences between the non-aMCI-AD and aMCI-AD groups. Bold denotes statistical significance.

**Table 2:** Cox regression models for aMCI–AD incidence using the PRS as a scale variable and a trichotomous variable. Models were adjusted for age, sex, and years of education, PC1, PC2, and ApoE ε4 genotype. Subgroup analyses abide by the same approach.

Total Sample		
PRShp ^1^	HR ^2^ (95% CI ^3^)	*p*-Value
PRS (scale)	**1.46 (1.14–1.86)**	**0.002**
PRS strata using quartiles		**0.021**
High	**2.81 (1.34–5.90)**	**0.006**
Intermediate	1.85 (0.91–3.77)	0.091
Low	Reference	
	**Men** (N = 258)	**Women** (N = 361)
**PRShp ^1^**	**HR ^2^ (95% CI ^3^), *p*-value**	**HR ^2^ (95% CI ^3^), *p*-value**
PRS (scale)	1.18 (0.78–1.78), 0.442	**1.60 (1.17–2.19), 0.003**
PRS strata using quartiles	0.216	0.103
High	2.38 (0.72–7.84), 0.154	**2.89 (1.09–7.64), 0.033**
Intermediate	1.28 (0.42–3.91), 0.664	2.13 (0.82–5.50), 0.119
Low	Reference	Reference
	**Younger than 72.67 years ** (N = 310)	**Older than 72.67 years ** (N = 309)
**PRShp ^1^**	**HR ^2^ (95% CI ^3^), *p*-value**	**HR ^2^ (95% CI ^3^), *p*-value**
PRS (scale)	**1.87 (1.21–2.90), 0.005**	1.31 (0.96–1.78), 0.089
PRS strata using quartiles	0.087	0.162
High	**3.71 (1.09–12.68), 0.037**	2.52 (0.97–6.54), 0.058
Intermediate	1.88 (0.59–6.02), 0.289	1.86 (0.74–4.70), 0.187
Low	Reference	Reference

^1^ polygenic risk score for hippocampal atrophy; ^2^ hazard ratio; ^3^ confidence interval. Bold denotes statistical significance. For each subgroup the number of events and subgroup size is provided.

**Table 3:** GEE (generalized estimating equations)-predicted rates of cognitive decline in cognitively unimpaired older adults. For high and intermediate PRSs by time interactions, the reference group was the low PRS by time product. Models were adjusted for age, sex, and years of education, PC1, PC2, ApoE4 genotype, and incidence of aMCI at follow-up.

Parameter	PRShp ^1^ (Scale) by Time Interaction (β ^2^, 95% CI ^3^, *p*-Value)	High PRShp ^1^ by Time Interaction (β ^2^, 95% CI ^3^, *p*-Value)	Intermediate PRShp ^1^ by Time Interaction (β ^2^, 95% CI ^3^, *p*-Value)
Global cognition	**−0.013 (−0.025, −0.000), 0.043**	**−0.038 (−0.075, −0.002), 0.038**	−0.017 (−0.045, 0.011), 0.236
Memory	−0.015 (−0.032, 0.001), 0.069	**−0.051 (−0.096, 0.006), 0.025**	−0.011 (−0.052, 0.030), 0.606
Visuospatial	−0.003 (−0.028, 0.021), 0.791	−0.024 (−0.091, 0.043), 0.486	−0.015 (−0.070, 0.040), 0.586
Executive	−0.012 (−0.025, 0.001), 0.079	−0.033 (−0.069, 0.002), 0.067	−0.031 (−0.063, 0.002), 0.066
Language	−0.012 (−0.025, 0.001), 0.076	−0.022 (−0.062, 0.017), 0.269	−0.010 (−0.046, 0.026), 0.588
Attention	−0.007 (−0.032, 0.017), 0.556	−0.010 (−0.082, 0.061), 0.780	−0.026 (−0.088, 0.036), 0.414

^1^ polygenic risk score for hippocampal atrophy; ^2^ regression coefficient;^, 3^ confidence interval. Bold denotes statistical significance.

## Data Availability

The data that support the findings of this study are available from the corresponding author [N.S.], unconditionally. Specific research questions and protocols have to be submitted for approval to grant access to the data of the HELIAD cohort for research purposes.

## Supplementary Materials

The following supporting information can be downloaded at: https://www.mdpi.com/article/10.3390/geriatrics10010014/s1. S1.1. Genotyping and Imputation in HELIAD. S1.2. Polygenic Risk Calculation. Table S1. Unadjusted relationship between PRS at different p-value thresholds (P_T_) and the risk of aMCI–AD. Table S2. GEE (generalized estimating equations)-predicted rates of cognitive decline in cognitively unimpaired older men vs. women. Models were adjusted for age, years of education, PC1, PC2, ApoE ε4 genotype and incidence of aMCI at follow-up. Table S3. GEE (generalized estimating equations)-predicted rates of cognitive decline in cognitively unimpaired older (≥65 years) adults over vs. under 74.42 years. Models were adjusted for sex, years of education, PC1, PC2, ApoE4 genotype, and incidence of amnestic MCI at follow-up [40,41,42,43,44,45,46,47,48].
