# Supplementary material for: Genetic Predisposition to Hippocampal Atrophy and Risk of Amnestic Mild Cognitive Impairment and Alzheimer’s Dementia
Source: Geriatrics (Basel). 2025 Jan 16;10(1):14. doi: 10.3390/geriatrics10010014 (PMC11755629; doi:10.3390/geriatrics10010014)
Supplement: Supplementary file 1 [file geriatrics-10-00014-s001.zip › geriatrics-3290278-supplementary.pdf]

Supplementary Materials

S1.1. Genotyping and Imputation in HELIAD

Genome-wide genotyping was performed at Life & Brain facilities (Germany) using the Illumina Infinium Global Screening Array and calling was generated by the "centre national de recherche en génétique humaine" (France) using the data generated by the centres involved in genotyping (Life & Brain, CNRGH and Amsterdam). Briefly, variants included in the removal marker list by Illumina were excluded and only variants for which the full-length probes aligned uniquely on GRCh38 genome without mismatches were kept. Variant intensity quality control was conducted for all autosomal variants, according to established thresholds, while sex-check was performed using chrX variants [1]. Samples with missingness > 0.05, sex inconsistencies or with heterozygosity rate that deviated > ± 6 Standard Deviation (SD) from the mean, were excluded.

To identify population outliers, we run Principal Component Analysis, using as reference dataset the 1000 Genome population and the combined dataset was projected onto two dimensions, using flashPCA2 [2]. To control for cryptic relatedness, we excluded one individual from each pair of samples with a kinship coefficient more than 0.125 (cut-off for second-degree relatives), yielding a final sample size of 1251 unrelated individuals. We excluded variants with a missingness > 0.05 in at least one genotyping center or having a differential missingness test P<10-10. The Hardy-Weinberg equilibrium test (p<10-6) was performed only in controls.

To improve the accuracy of imputation, we compared the frequencies of variants (chi-squared test) against two reference panels, the non-Finnish population of the Haplotype Reference Consortium r1.1 (HRC) [3] and the non-Finnish population of Genome Aggregation Database v3 (gnomAD) [4]. Variants showing a x2 >3,000 in both HRC and gnomAD or in one reference panel without being present in the other were excluded. Finally, GWASs were performed between controls across genotyping centers to assess frequency differences between genotyping centers, using SNPTEST [5], under an additive model and adjusting on associated Principal Components. Variants with a Likelihood Ratio Test of p < 10-5 were excluded. Furthermore, we removed ambiguous variants with Minor Allele Frequency (MAF) > 0.4 and we kept only one copy of any duplicated variants, prioritizing the one with the lowest missingness. All qualified samples and variants were imputed on Michigan Imputation Server (v1.2.4) [6], using the TOPMed Freeze 5 reference panel. Phasing and imputation were performed using EAGLE v2.4 [7] and Minimac4 v4-1.0.2 software, respectively.

S1.2. Polygenic Risk Calculation

In the HELIAD genotype data and prior to PRS calculation, imputed SNPs dosages for a total of 5,611,082 SNPs, with MAF>0.05, call rate >95% and imputation quality score >0.4 were converted to best-guess genotypes. The PRSice software (http://prsice.info/) [8] was utilized to construct PRS for each HELIAD participant applying the clumping and thresholding (C+T) method, following the approach originally described by the International Schizophrenia Consortium [9]. In particular, the risk score of each SNP is calculated by multiplying the risk allele number (0, 1, 2) with the corresponding effect size for the reference allele reported in the GWAS summary data. Effect size is used as a weight of the risk that each SNP confers and the PRS for each individual is computed as the sum of log(OR)-weighted genotypes of all SNPs.

To ensure that only independent markers are included in the computed PRS score, we first clumped SNPs of the HELIAD data for linkage disequilibrium (SNPs with r2=0.1 in 250 kb-windows were removed). Markers within the major histocompatibility complex (MHC) LD region on chromosome 6 (chr6:27-33Mb, hg19) were also excluded from PRS computation process due to the high polymorphic nature of this region. Moreover, ApoE region, defined as 1MB up and downstream of the gene (chr19: 44,409,039-46,412,650) was excluded from calculation of the scores and ApoE4 genotype was added as covariate in statistical models.

Supplementary Table S1. Unadjusted relationship between PRS at different P-thresholds (P_T) and the risk of aMCI / AD.

PRShp[1] P_T[2]	Number of SNPs[3]	Hazard Ratio	P-value	AUC[4]
5e-8	11	1.087	0.486	0.657
5e-05	139	1.118	0.329	0.658
0.0001	229	1.122	0.332	0.660
0.001	1321	1.224	0.096	0.668
**0.01**	**8475**	**1.387**	**0.005**	**0.689**
0.05	28263	1.275	0.051	0.666
**0.1**	**45101**	**1.286**	**0.038**	**0.681**
0.2	68809	1.210	0.114	0.673
0.3	85315	1.159	0.218	0.672
0.4	97744	1.143	0.259	0.668
0.5	107514	1.141	0.263	0.667
1	135145	1.145	0.267	0.668

[1] Polygenic risk score for hippocampal atrophy, [2] p-value threshold, [3] Single nucleotide polymorphism, [4] Area under the curve estimated using logistic regression models with the presence of AD-aMCI as the outcome, and the different PRS thresholds as the main predictors. **Bold** denotes statistical significance.

Supplementary Table S2. GEE (generalized estimating equations) - predicted rates of cognitive decline in cognitively unimpaired older men vs. women. Models were adjusted for age, years of education, PC1, PC2, ApoE ε4 genotype and incidence of aMCI at follow-up

**Parameter**	**PRShp[1] by time in men (β[2], 95% CI[3], p-value)**	**PRShp[1] by time in women (β[2], 95% CI[3], p-value)**
Global cognition	0.008 (-0.012, 0.028), 0.444	**-0.026 (-0.041, -0.011), 0.001**
Memory	-0.001 (-0.025, 0.024), 0.963	**-0.025 (-0.047, -0.003), 0.024**
Visuospatial	0.039 (-0.005, 0.082), 0.080	**-0.030 (-0.057, -0.002), 0.033**
Executive	0.007 (-0.015, 0.028), 0.544	**-0.023 (-0.039, -0.008), 0.003**
Language	0.005 (-0.017, 0.028), 0.659	**-0.023 (-0.039, -0.007), 0.006**
Attention	0.014 (-0.022, 0.051), 0.442	-0.022 (-0.053, 0.009), 0.166

[1] Polygenic risk score, [2] Regression coefficient, [3] Confidence interval.
Bold denotes statistical significance.

Supplementary Table S3. GEE (generalized estimating equations) -predicted rates of cognitive decline in cognitively unimpaired older (≥65 years) adults over vs. under 74.42 years. Models were adjusted for sex, years of education, PC1, PC2, ApoE4 genotype and incidence of amnestic MCI at follow-up.

Parameter	PRShp[1] by time in those younger than 72.67 years (β[2], 95% CI[3], p-value)	PRShp[1] by time in those older than 72.67 years (β[2], 95% CI[3], p-value)
Global cognition	-0.012 (-0.026, 0.003), 0.123	-0.015 (-0.036, 0.006), 0.161
Memory	-0.009 (-0.032, 0.014), 0.439	-0.020 (-0.044, 0.005), 0.111
Visuospatial	-0.009 (-0.039, 0.021), 0.568	-0.005 (-0.045, 0.035), 0.809
Executive	**-0.017 (-0.034, -0.000), 0.044**	-0.007 (-0.028, 0.014), 0.520
Language	-0.011 (-0.029, 0.006), 0.201	-0.012 (-0.032, 0.009), 0.260
Attention	-0.007 (-0.033, 0.018), 0.580	-0.006 (-0.051, 0.040), 0.809

[1] Polygenic risk score, [2] Regression coefficient, [3] Confidence interval.
Bold denotes statistical significance.

References

1. Grove, M.L.; Yu, B.; Cochran, B.J.; Haritunians, T.; Bis, J.C.; Taylor, K.D.; Hansen, M.; Borecki, I.B.; Cupples, L.A.; Fornage, M.; et al. Best Practices and Joint Calling of the HumanExome BeadChip: The CHARGE Consortium. *PLoS ONE* **2013**, *8*, e68095.
2. Abraham, G.; Qiu, Y.; Inouye, M. FlashPCA2: Principal component analysis of Biobank-scale genotype datasets. *Bioinformatics* **2017**, *33*, 2776–2778.
3. McCarthy, S.; Das, S.; Kretzschmar, W.; Delaneau, O.; Wood, A.R.; Teumer, A.; Kang, H.M.; Fuchsberger, C.; Danecek, P.; Sharp, K.; et al. A reference panel of 64,976 haplotypes for genotype imputation. *Nat Genet.* **2016**, *48*, 1279–1283.
4. Karczewski, K.J.; Francioli, L.C.; Tiao, G.; Cummings, B.B.; Alföldi, J.; Wang, Q.; Collins, R.L.; Laricchia, K.M.; Ganna, A.; Birnbaum, D.P.; et al. The mutational constraint spectrum quantified from variation in 141,456 humans. *Nature* **2020**, *581*, 434–443.
5. Marchini, J.; Howie, B.; Myers, S.; McVean, G.; Donnelly, P. A new multipoint method for genome-wide association studies by imputation of genotypes. *Nat Genet.* **2007**, *39*, 906–913.
6. Das, S.; Forer, L.; Schönherr, S.; Sidore, C.; Locke, A.E.; Kwong, A.; Vrieze, S.I.; Chew, E.Y.; Levy, S.; McGue, M.; et al. Next-generation genotype imputation service and methods. *Nat. Genet.* **2016**, *48*, 1284–1287. https://doi.org/10.1038/ng.3656.
7. Loh, P.R.; Danecek, P.; Palamara, P.F.; Fuchsberger, C.; AReshef, Y.; KFinucane, H.; Schoenherr, S.; Forer, L.; McCarthy, S.; Abecasis, G.R.; et al. Reference-based phasing using the Haplotype Reference Consortium panel. *Nat Genet.* **2016**, *48*, 1443–1448.
8. Choi, S.W.; O'Reilly, P.F. PRSice-2: Polygenic Risk Score software for biobank-scale data. *Gigascience* **2019**, *8*, giz082.
9. International Schizophrenia Consortium; Purcell, S.M.; Wray, N.R.; Stone, J.L.; Visscher, P.M.; O'Donovan, M.C.; Sullivan, P.F.; Sklar, P. Common polygenic variation contributes to risk of schizophrenia and bipolar disorder. *Nature* **2009**, *460*, 748–752.
